# Analysis of multidrug efflux transporters in resistance to fatty acid salts reveals a TolC-independent function of EmrAB in *Salmonella enterica*

**DOI:** 10.1371/journal.pone.0266806

**Published:** 2022-04-14

**Authors:** Tomohiro Yoneda, Hiroki Sakata, Seiji Yamasaki, Mitsuko Hayashi-Nishino, Kunihiko Nishino

**Affiliations:** 1 SANKEN (The Institute of Scientific and Industrial Research), Osaka University, Ibaraki, Osaka, Japan; 2 Graduate School of Pharmaceutical Sciences, Osaka University, Suita, Osaka, Japan; 3 Institute for Advanced Co-Creation Studies, Osaka University, Suita, Osaka, Japan; 4 Center for Infectious Disease Education and Research, Osaka University, Suita, Osaka, Japan; University of Cambridge, UNITED KINGDOM

## Abstract

Fatty acids salts exhibit bacteriostatic and bactericidal effects to inhibit bacterial growth and survival. Bacteria adapt to their environment to overcome these antibacterial effects through undefined mechanisms. In Gram-negative bacteria, drug efflux systems are associated with resistance to various substances. Studies have identified multiple drug efflux systems in *Salmonella enterica*. The aim of this study was to investigate whether drug efflux systems contribute to fatty acid salts resistance in *S*. *enterica*. We used deletion and overexpressing strains of *S*. *enterica* for drug efflux transporters. Susceptibility to fatty acid salts was determined by measuring minimum inhibitory concentrations and performing growth assays. Our findings revealed that *acrAB*, *acrEF*, *emrAB* and *tolC* in *S*. *enterica* contribute resistance to fatty acid salts. Furthermore, EmrAB, which is known to function with TolC, contributes to the fatty acid salts resistance of *S*. *enterica* in a TolC-independent manner. This study revealed that drug efflux systems confer fatty acid satls resistance to *S*. *enterica*. Notably, although EmrAB is normally associated with antimicrobial resistance in a TolC-dependent manner, it was found to be involved in fatty acid salts resistance in a TolC-independent manner, indicating that the utilization of TolC by EmrAB is substrate dependent *in S*. *enterica*.

## Introduction

Fatty acid salts which possess amphipathic properties, exhibit some antibacterial activity. In biological systems, fatty acid salts typically contain 4–28 carbon atoms [1]. Salts of fatty acids that contain <8, 8–12 and >12 carbon atoms are defined as short-, medium- and long-chain fatty acid salts, respectively [2]. The antimicrobial properties of several fatty acid salts were reported. Lauric acid and myristoleic acid, which are saturated fatty acids, possess strong antibacterial activity [3]. Several reports have demonstrated the inhibitory effects of fatty acid salts on microorganisms [4]. Fatty acid salts act as antibacterial agents mainly by destabilising bacterial cell membranes, which leads to increased cell permeability and cell lysis, thereby inhibiting bacterial cell growth. The mechanisms of antibacterial activity induced by fatty acid salts have been classified as follows: (1) increased membrane permeability and leakage, (2) disruption of the electron transport chain and uncoupling of oxidative phosphorylation and (3) inhibition of membrane enzymatic activities and nutrient uptake [2].

Some bacterial cells naturally resist the antibacterial action of fatty acid salts through several strategies. It was reported that the outer cell membranes of Gram-negative species protect against fatty acid salts [5]. Some bacteria possess outer cell membranes that are more highly charged and less hydrophobic. The change in cell-surface hydrophobicity makes fatty acid salts less attracted to bacterial cells and less likely to permeate the inner membranes of bacteria. In some bacterial strains, membrane-localised carotenoids may provide resistance against disruption by fatty acid salts. Carotenoids are antioxidants that can stabilise the cell membrane by decreasing its fluidity. Thus, carotenoids may counteract the effects of reactive degradation products of fatty acid salts or fatty acid salts-induced increase in membrane fluidity [6]. There is a need to elucidate the resistance mechanisms against antibacterial action by fatty acid salts to understand how certain bacteria evade or abrogate their bactericidal effects [7].

Multidrug efflux transporters cause serious problems in cancer chemotherapy and in the treatment of bacterial infections. In bacteria, resistance to various compounds is often associated with multidrug efflux transporters that decrease cellular drug accumulation. Efflux transporters are classified into the following six families based on sequence similarity: major facilitator (MF); resistance-nodulation-cell division (RND); small multidrug resistance (SMR); multidrug and toxic compound extrusion (MATE); ATP-binding cassette (ABC); and proteobacterial antimicrobial compound efflux (PACE). The determination of bacterial genome sequences enables us to trace putative drug resistance genes in Gram-negative bacteria, including *Salmonella enterica* serovar Typhimurium [8].

Efflux transporters prevent intracellular accumulation of bile salts and fatty acids [9–13]. Consistently, for some bacteria multidrug efflux transporters are hypothesised to play a key role in overcoming the antibacterial effect of fatty acid salts. We evaluated the physiological functions of multidrug efflux transporters in resistance to fatty acid salts by using various strains of *S*. *enterica* deficient or overexpressing genes encoding multidrug efflux transporters. This analysis helped to identify multidrug efflux transporters and mechanisms involved in bacterial resistance to fatty acid salts.

## Materials and methods

### Bacterial strains, plasmids and growth conditions

The bacterial strains and plasmids used in this study are listed in Table 1. The *S*. *enterica* serovar Typhimurium strains were derived from the wild-type strain ATCC 14028s [14]. The *E*. *coli* strains were derived from the wild-type strain MG1655 [15]. Bacterial strains were grown at 37°C in Lysogeny Broth (LB) with appropriate antibiotics when necessary [16].

**Table 1:** *Salmonella enterica* and *Escherichia coli* strains used in this study.

Strains		Characteristics	Source or reference
***S*. *enterica***			
	ATCC 14028s	*S*. *enterica* serovar Typhimurium wild-type	[8]
	NKS175	Δ*acrAB*	[8]
	NKS181	Δ*acrAB*Δ*acrEF*	[8]
	NKS183	Δ*acrAB*Δ*acrEF*Δ*acrD*	[8]
	NKS185	Δ*acrAB*Δ*acrEF*Δ*acrD*Δ*mtdABC*	[8]
	NKS186	Δ*acrAB*Δ*acrEF*Δ*acrD*Δ*mtdABC*Δ*mdsABC*::Cm^R^	[8]
	NKS188	Δ*acrAB*Δ*acrEF*Δ*acrD*Δ*mtdABC*Δ*mdsABC*Δ*emrAB*::Cm^R^	[8]
	NKS190	Δ*acrAB*Δ*acrEF*Δ*acrD*Δ*mtdABC*Δ*mdsABC*Δ*emrAB*::Cm^R^ Δ*mdfA*::Km^R^	[8]
	NKS195	Δ*acrAB*Δ*acrEF*Δ*acrD*Δ*mtdABC*Δ*mdsABC*Δ*emrAB*Δ*mdfA*Δ*mdtK*::Cm^R^	[8]
	NKS196	Δ*acrAB*Δ*acrEF*Δ*acrD*Δ*mtdABC*Δ*mdsABC*Δ*emrAB*Δ*mdfA*Δ*mdtK*::Cm^R^ Δ*macAB*::Km^R^	[8]
	NKS174	Δ*tolC*	[8]
	NKS133	Δ*emrAB*::Cm^R^	This study
	NKS825	Δ*tolC*Δ*emrAB*::Cm^R^	This study
	NKS845	Δ*tolC*Δ*emrAB*::Cm^R^*/*vector (pUC118)	This study
	NKS846	Δ*tolC*Δ*emrAB*::Cm^R^*/*p*emrAB*	This study
	NKS148	Δ*acrB*::Km^R^	[8]
	NKS442	Δ*acrB*::Km^R^/vector (pUC118)	[8]
	NKS773	Δ*acrB*::Km^R^/p*acrAB*	[8]
	NKS757	Δ*acrB*::Km^R^/p*acrD*	[8]
	NKS756	Δ*acrB*::Km^R^/p*acrEF*	[8]
	NKS484	Δ*acrB*::Km^R^/p*mdsAB*	[8]
	NKS758	Δ*acrB*::Km^R^/p*mdtABC*	[8]
	NKS443	Δ*acrB*::Km^R^/p*emrAB*	[8]
	NKS759	Δ*acrB*::Km^R^/p*mdfA*	[8]
	NKS447	Δ*acrB*::Km^R^/p*mdtK*	[8]
	NKS446	Δ*acrB*::Km^R^/p*macAB*	[8]
	EG15129	Δ*emrAB*–*lacZY*^+^ Km^R^	[8]
***E*. *coli***			
	MG1655	*Escherichia coli* wild-type	[15]
	NKE348	Δ*acrAB*	[17]
	NKE473	Δ*acrAB*/vector (pHSG399)	[17]
	NKE393	Δ*acrAB*/p*emrAB*	[17]

### Construction of gene deletion mutants

To construct gene deletion mutants of *S*. *enterica* and *E*. *coli*, gene disruption was performed as described by Datsenko and Wanner [8, 18]. The chloramphenicol resistance *cat* gene or the kanamycin resistance *aph* gene, flanked by Flp recognition sites, was PCR amplified and the products were used to transform the recipient ATCC 14028s or MG1655 strain harbouring plasmid pKD46, which expresses the Red recombinase. The chromosomal structures of the mutated loci were verified by PCR and *cat* and *aph* were eliminated using plasmid pCP20 [18].

### Plasmid construction

The plasmids carrying *acrAB*, *acrD*, *acrEF*, *mdtABC*, *mdsABC*, *emrAB*, *mdfA*, *mdtK* or *macAB* in *S*. *enterica* were constructed as described [8, 19, 20]. The plasmids carrying *emrAB*, gene in *E*. *coli* were constructed as described [17].

### Determination of minimum inhibitory concentrations of toxic compounds

Antibacterial activities of various agents were determined on LB agar plates containing sodium hexanoate (C6), sodium octanoate (C8), sodium decanoate (C10) and sodium dodecanoate (C12) (Sigma-Aldrich, St Louis, MO, USA) at various concentrations. Agar plates were prepared using the 2-fold agar dilution technique [21]. To determine minimum inhibitory concentrations (MICs), bacteria were grown in LB at 37°C overnight, diluted with the same medium and then tested at a final inoculum concentration of 10^5^ cfu/μL using a multipoint inoculator (Sakuma Seisakusyo, Tokyo, Japan) after incubation at 37°C for 20 h. MIC was the lowest concentration of the compound required to inhibit cellular growth.

### β-galactosidase assay

Single colonies of each bacterial strain were inoculated into 2 mL LB medium containing antibiotics. After overnight incubation at 37°C, the cultures were diluted 1:50 in LB medium. The cells were then incubated at 37°C until they reached an OD_600_ of 0.8. To examine the effect of fatty acid salts on gene expression, 20 μg/mL sodium dodecanoate was added to secondary cultures. β-galactosidase activity in cell lysates was assayed using o-nitrophenyl-β-D-galactopyranoside as a substrate, as described by Miller [22].

### Measurement of bacterial growth

Single colonies of each bacterial strain were inoculated into 2 mL LB. Bacterial cells were cultured overnight at 37°C; then, 100 μL cell cultures were diluted in 5 mL of the same medium. The diluted bacterial cells were incubated at 37°C until OD_600_ reached 0.5. Then, the bacterial cells were diluted in the same medium to an OD_600_ of 0.05 and incubated in NUNC Edge 96-well plates (Thermo Scientific, MA, USA) with shaking at 37°C for 7 h. Bacterial growth was monitored using the Infinite M200 PRO plate reader (Tecan, Männedorf, Switzerland). To assay the effects of toxic compounds on cell growth, 40–50 μg/mL sodium dodecanoate, 8 μg/mL nalidixic acid, 1 μg/mL novobiocin and 100 μg/mL bile salt were added to the secondary cultures.

## Results

### Susceptibility of multidrug efflux transporter-deficient or -overexpressing strains to various fatty acid salts

To evaluate the involvement of multidrug efflux transporters in *S*. *enterica* against resistance to fatty acid salts, we investigated the susceptibility of multidrug efflux transporter-deficient or -overexpressing strains by measuring MICs of sodium hexanoate (C6), sodium octanoate (C8), sodium decanoate (C10) and sodium dodecanoate (C12). Fatty acid salts with 6–12 carbon atoms were used because salts of fatty acids with >14 carbon atoms are difficult to dissolve in the medium. The MIC results indicate that the antibacterial activity of fatty acid salts increases with the number of carbon atoms (Table 2). For example, the results show that the MIC values for Δ*tolC* in *S*. *enterica* become lower as the number of carbon atoms increases (Table 2).

**Table 2:** Susceptibility of *S*. *enterica* and *E*. *coli* strains to sodium hexanoate (C6), sodium octanoate (C8), sodium decanoate (C10) and sodium dodecanoate (C12).

	MIC (μg/ml)			
	C6	C8	C10	C12
***S*. *enterica***				
Wild-type	10000	10000	10000	> 5000
Δ*acrAB*	10000	10000	1250	1250
Δ*acrAB*Δ*acrEF*	10000	10000	1250	1250
Δ*acrAB*Δ*acrEF*Δ*acrD*	10000	10000	1250	1250
Δ*acrAB*Δ*acrEF*Δ*acrD*Δ*mtdABC*	10000	10000	1250	1250
Δ*acrAB*Δ*acrEF*Δ*acrD*Δ*mtdABC*Δ*mdsABC*	10000	10000	1250	1250
Δ*acrAB*Δ*acrEF*Δ*acrD*Δ*mtdABC*Δ*mdsABC*Δ*emrAB*	10000	10000	313	39
Δ*acrAB*Δ*acrEF*Δ*acrD*Δ*mtdABC*Δ*mdsABC*Δ*emrAB*Δ*mdfA*	10000	10000	313	39
Δ*acrAB*Δ*acrEF*Δ*acrD*Δ*mtdABC*Δ*mdsABC*Δ*emrAB*Δ*mdfA*Δ*mdtK*	10000	10000	313	39
Δ*acrAB*Δ*acrEF*Δ*acrD*Δ*mtdABC*Δ*mdsABC*Δ*emrAB*Δ*mdfA*Δ*mdtK*Δ*macAB*	10000	10000	313	39
Δ*tolC* ^*a*^	10000	2500	313	156
Δ*emrAB*	10000	10000	10000	> 5000
Δ*tolC*Δ*emrAB* ^*b*^	5000	2500	156	20
Δ*tolC*Δ*emrAB/*vector ^*c*^	5000	2500	156	20
Δ*tolC*Δ*emrAB/*p*emrAB* ^*d*^	5000	2500	1250	156
Δ*acrB*	10000	5000	625	625
Δ*acrB*/vector	10000	5000	625	625
Δ*acrB*/p*acrAB*	10000	5000	2500	5000
Δ*acrB*/p*acrD*	10000	10000	1250	2500
Δ*acrB*/p*acrEF*	10000	10000	2500	5000
Δ*acrB*/p*mdsAB*	10000	5000	1250	625
Δ*acrB*/p*mdtABC*	10000	5000	1250	625
Δ*acrB*/p*emrAB*	10000	5000	2500	5000
Δ*acrB*/p*mdfA*	10000	10000	1250	2500
Δ*acrB*/p*mdtK*	10000	5000	625	625
Δ*acrB*/p*macAB*	10000	5000	625	625
***E*. *coli***				
Wild-type	20000	10000	10000	> 5000
Δ*acrAB*	5000	5000	1250	625
Δ*acrAB*/vector	5000	5000	1250	625
Δ*acrAB*/p*emrAB*	5000	5000	2500	5000

MIC determinations were repeated at least three times.

MIC values of deoxycholic acid sodium salt were > 40000 μg/ml for the wild-type strain, 156 μg/ml for aΔtolC, 39 μg/ml for bΔtolCΔemrAB and cΔtolCΔemrAB/vector, and 156 μg/ml for dΔtolCΔemrAB/pemrAB.

In *S*. *enterica*, the deletion of *acrAB* resulted in strains with increased susceptibility to sodium decanoate and sodium dodecanoate. When *emrAB* was deleted from the Δ*acrAB*Δ*acrEF*Δ*acrD*Δ*mdtABC*Δ*mdsABC* mutant, the resulting strain exhibited increased susceptibility to sodium decanoate and sodium dodecanoate (Table 2). On the other hand, the single deletion of *emrAB* revealed no apparent change of the susceptibility to fatty acid salts compared with wild-type stain in *S*. *enterica*. It is implicated that the contribution of EmrAB to the resistance to sodium decanoate and dodecanoate in the *acrAB*-deleted mutant because constitutively expressed AcrAB masks the effect of EmrAB. The strain lacking *tolC* was sensitive to sodium octanoate, sodium decanoate and sodium dodecanoate more than Δ*acrAB*. Interestingly, the *tolC emrAB* double mutant was more susceptible than the *tolC* single mutant (Table 2) whereas it is known that EmrAB function with TolC. Overexpression of *emrAB* conferred resistance to the *tolC emrAB* double mutant against sodium decanoate and sodium dodecanoate. Plasmids carrying *acrAB*, *acrEF*, or *emrAB* conferred 4- and 8-fold higher resistance to the *acrB* mutant against sodium decanoate and sodium dodecanoate, respectively. Overexpression of *acrD* or *mdfA* in the *acrB* mutant resulted in 4-fold increase in resistance to sodium dodecanoate. Similarly, when *emrAB* was overexpressed in the *acrAB* deficient strain in *E*. *coli*, 8-fold increased resistance to sodium dodecanoate was observed (Table 2). In the following section, we focused on *emrAB* of *S*. *enterica* because it largely contributes to fatty acid salts resistance both when it is deleted and expressed.

### Activation of the *emrAB* promoter by fatty acid salts

**Fig 1:**
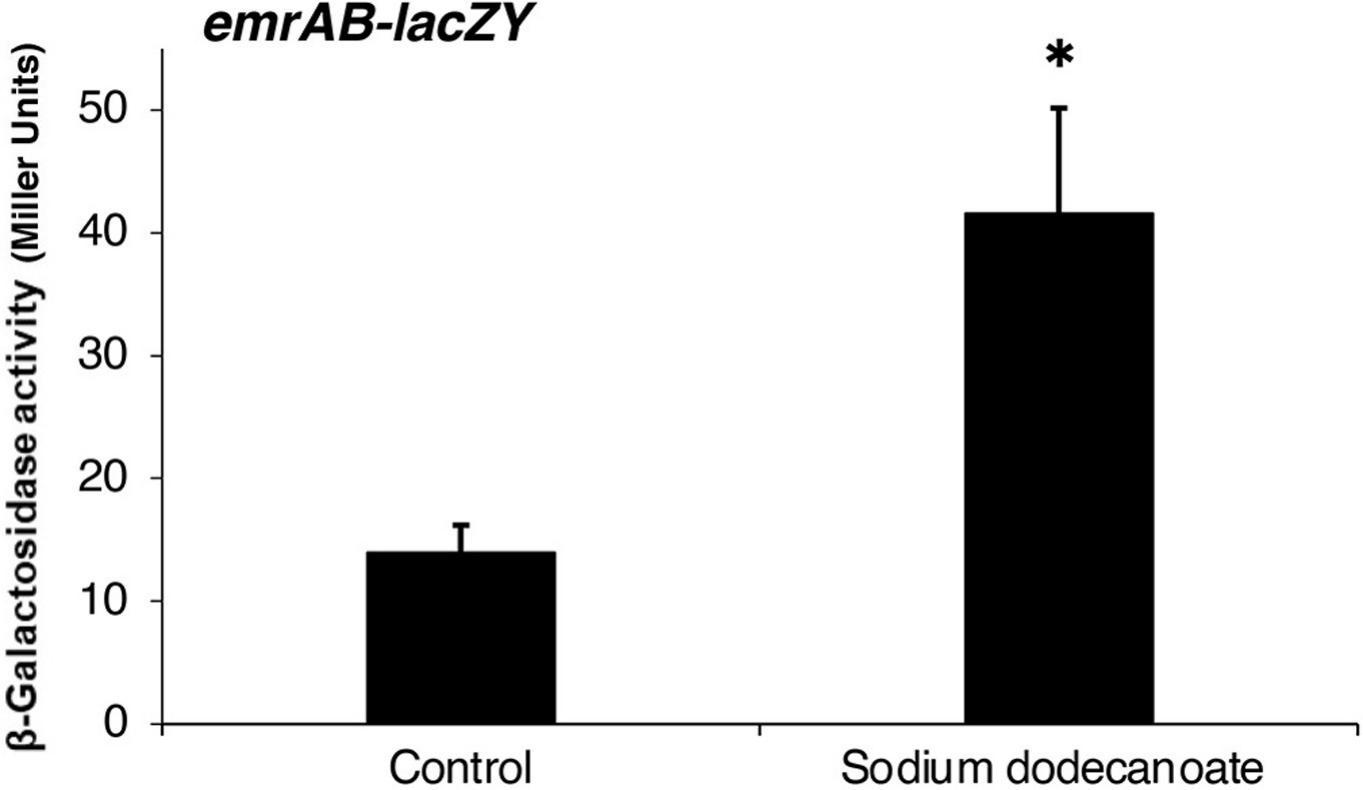
Effect of the fatty acid salt on the promoter activity of *emrAB*. β-galactosidase activity in *S*. *enterica* strain in which *lacZY* genes replaced the chromosomal copy of *emrAB* grown with or without sodium dodecanoate (C12). Activities of EG15129 were determined as described in Materials and Methods. The value displayed correspond to mean values of five independent experiments. Error bars correspond to the standard deviation. Student’s *t*-test; *, P < 0.01 versus control.

Our findings suggest that *emrAB* confers resistance to sodium decanoate and sodium dodecanoate; however, whether fatty acid salts induce the expression of *emrAB* is unknown. In the previous study, it was suggested that *emrAB* expression needs to be induced by additional cues because the promoter activity of *emrAB* is not high as that of constitutively expressed *acrAB* under laboratory conditions [8]. In *E*. *coli*, it was previously reported that CCCP, nalidixic acid and other chemicals induce the expression of *emrAB* [23]. To investigate whether the expression of *emrAB* is regulated by sodium dodecanoate in *S*. *enterica*, we cultured the *S*. *enterica* strain in which the *lacZY* genes replaced the chromosomal copy of *emrAB*, with or without sodium dodecanoate. Then, the promoter activity of *emrAB* was evaluated using the β-galactosidase assay (Fig 1). The results revealed that *emrAB* is transcriptionally activated by sodium dodecanoate—3-fold higher than in the absence of fatty acid salts.

### Effect of *emrAB* deletion on the *S*. *enterica* growth in the presence of sodium dodecanoate

**Fig 2:**
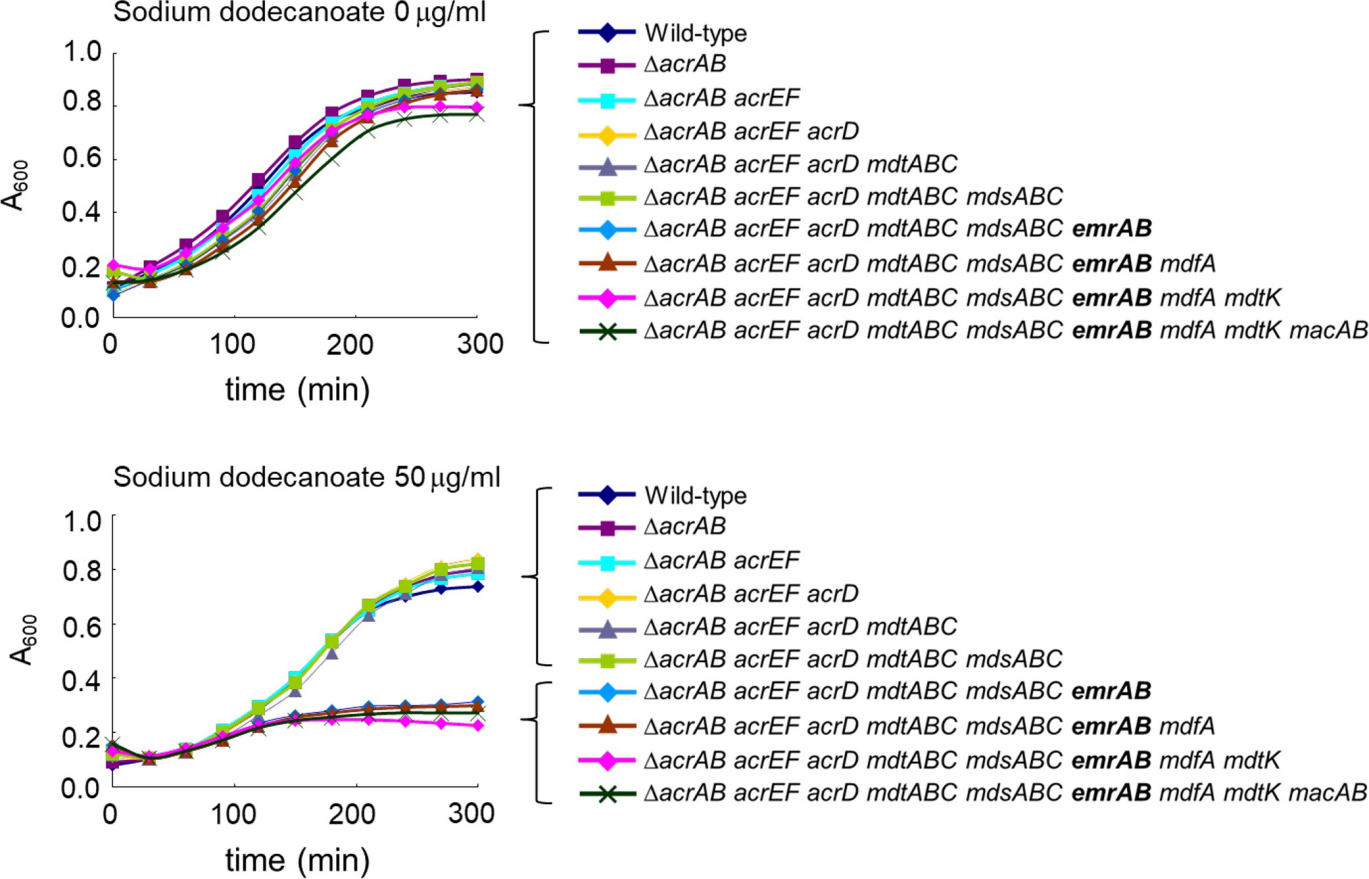
Effect of sodium dodecanoate on the growth of *Salmonella enterica*. The growth of *S*. *enterica* with stepwise deletion of multidrug efflux transporter genes was measured with or without sodium dodecanoate. Shown is the result of one of the three experiments, which gave similar results.

The MIC results revealed that sodium dedecanoate has the higher antibacterial activity than other fatty acid salts tested. To confirm the importance of *emrAB* role on the sodium dodecanoate resistance, the bacterial growth was measured in the presence of sodium dodecanoate with several *S*. *enterica* strain lacking multidrug efflux transporters (Fig 2). When *emrAB* was deleted from the Δ*acrAB*Δ*acrEF*Δ*acrD*Δ*mtdABC*Δ*mdsABC*, the mutant was inhibited by 50 μg/ml sodium dodecanoate whereas the mutant grew as the wild type strain without sodium dodecanoate. This is consistent with the MIC result. These data indicated that EmrAB contributes to the sodium dodecanoate intrinsic resistance of *S*. *enterica* where five efflux systems are deleted.

### TolC-independent contribution of EmrAB on sodium dodecanoate resistance

MIC results revealed that the susceptibility of *S*. *enterica* with the *emrAB* deletion from the Δ*acrAB*Δ*acrEF*Δ*acrD*Δ*mtdABC*Δ*mdsABC* mutant was higher than that of Δ*tolC* against sodium dodecanoate. The *tolC emrAB* double mutant was also more susceptible than the *tolC* single mutant to fatty acid salts (Table 2). This finding suggests that EmrAB functions in resistance to fatty acid salts without TolC.

**Fig 3:**
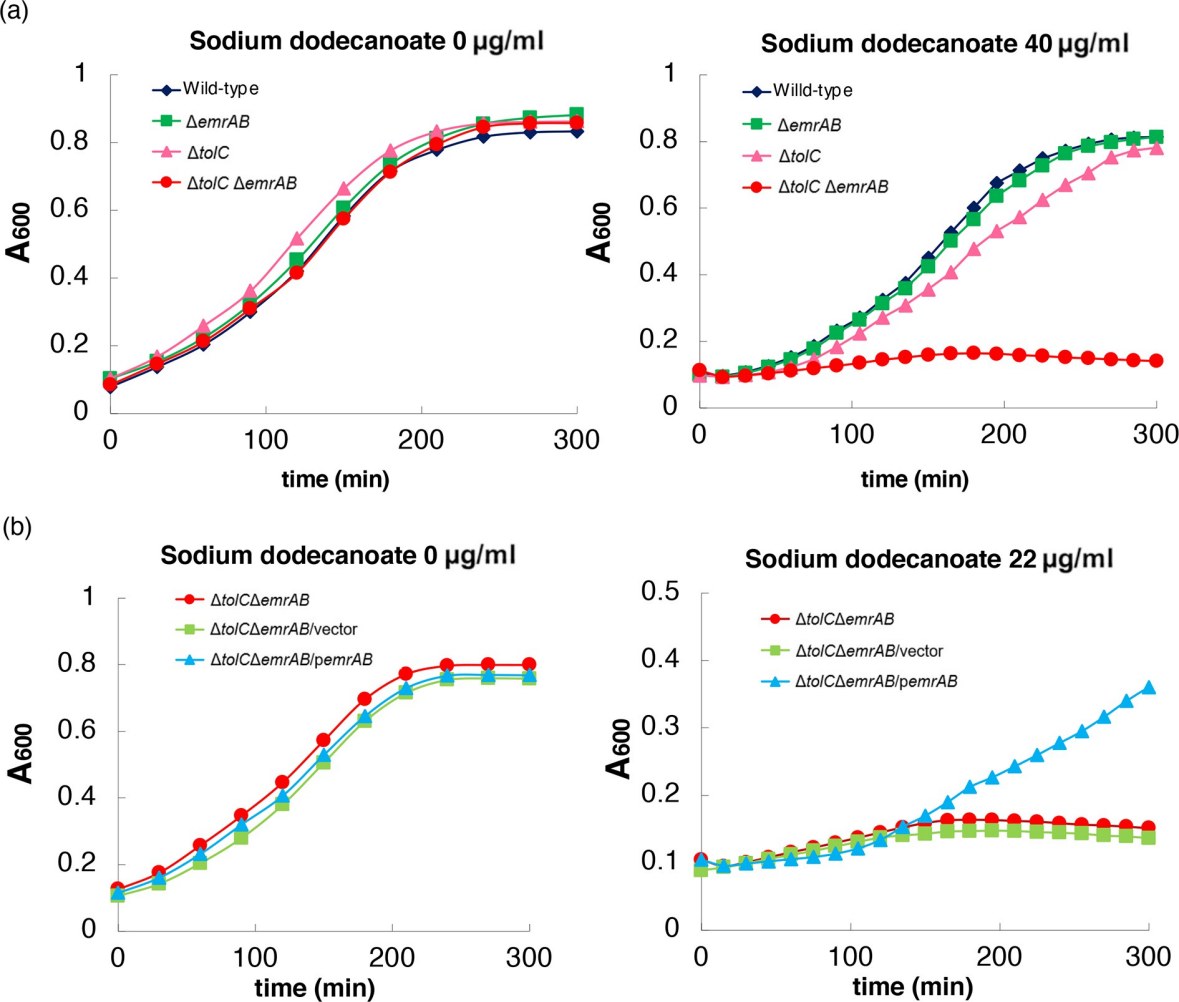
Effects of *tolC* and *emrAB* on the growth of *Salmonella enterica* in the presence of sodium dodecanoate. (a) Growth of the wild-type strain, *emrAB*, *tolC* and *tolC emrAB* mutants with or without sodium dodecanoate. (b) Growth of *tolC emrAB* mutant, *tolC emrAB* harbouring vector or p*emrAB* with or without sodium dodecanoate. Shown is the result of one of the three experiments, which gave similar results.

To confirm these findings, the growth of *S*. *enterica* Δ*tolC*, Δ*emrAB* and Δ*tolC*Δ*emrAB* mutants were measured with or without 40 μg/ml sodium dodecanoate (Fig 3A). Growth of all strains were same without sodium dodecanoate, however only the growth of Δ*tolC*Δ*emrAB* was inhibited in the presence of sodium dodecanoate (Fig 3A). This sensitivity was complemented when the plasmid carrying *emrAB* was transformed into the Δ*tolC*Δ*emrAB* mutant (Fig 3B). This finding indicates that EmrAB confer fatty acid salts resistance in TolC independent manner. The deletion of *emrAB* alone from the wild-type strain did not alter sodium dodecanoate sensitivity, suggesting that AcrAB, which is constitutively expressed and function with TolC, masks the function of EmrAB.

### Effect of deletion of drug efflux genes from the *tolC* mutant on the fatty acid salt resistance

**Fig 4:**
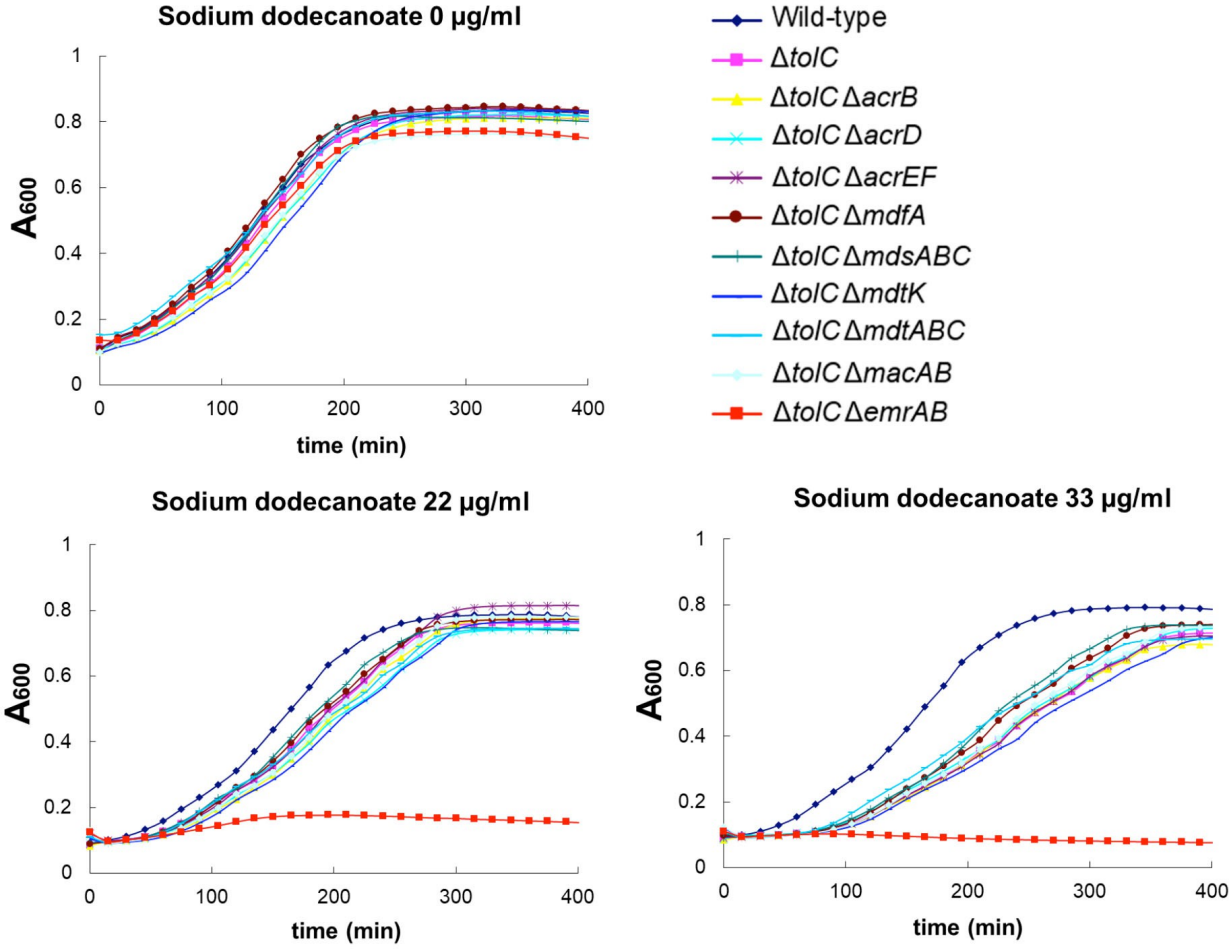
Effect of deletion of drug efflux genes from the *tolC* mutant on the growth of *S*. *enterica* in the presence of sodium dodecanoate. The growth of the wild-type and *tolC* mutant of *S*. *enterica* strains with the deletion of the multidrug efflux transporter gene were measured with or without sodium dodecanoate at concentrations indicated. Shown is the result of one of the three experiments, which gave similar results.

TolC works as a multifunctional outer membrane channel to form a complex with multiple drug efflux systems [20, 24]. The results above showed that the deletion of *emrAB* from Δ*tolC* made *S*. *enterica* be sensitive to sodium dodecanoate, indicating TolC-independent function of EmrAB to fatty acid salts resistance. To see whether similar effects are observed with other transporters, we examined the effects of deletion of *acrB*, *acrD*, *acrEF*, *mdfA*, *mdsABC*, *mdtK*, *mdtABC*, *macAB* or *emrAB* from the *tolC* mutant on the fatty acid salt resistance (Fig 4). All the deletion mutants grew as the wild-type strain without the fatty acid salt. Only the growth of the *tolC emrAB* double mutant was inhibited by sodium dodecanoate. By contrast, other double mutants and Δ*tolC* were grown in the presence of sodium dodecanoate (Fig 4), indicating the important role of EmrAB in the fatty acid salt resistance.

### TolC dependence of EmrAB on different substrates

**Fig 5:**
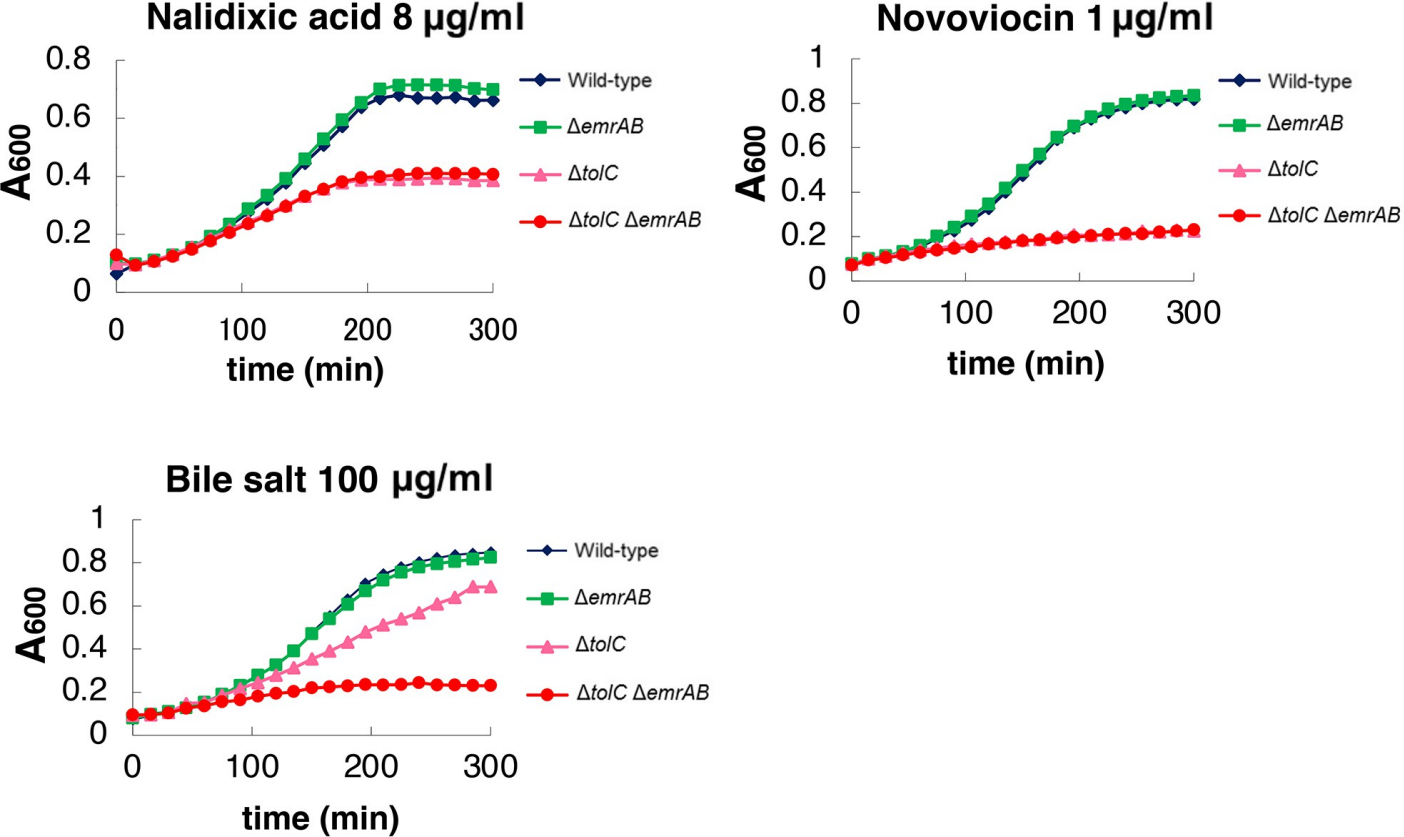
Different effect of the deletion of *tolC* and *emrAB* on the growth of *S*. *enterica* in presence of EmrAB substrates. Growth was measured in the presence of 1 μg/ml nalidixic acid, 8 μg/ml novobiocin, or 100 μg/ml bile salt. Shown is the result of one of the three experiments, which gave similar results.

The results in this study showed that EmrAB confers resistance to sodium dodecanoate in a TolC-independent manner. To identify the TolC-dependency of EmrAB for other substrates, we measured the growth of the wild-type, Δ*emrAB*, Δ*tolC* and Δ*tolC*Δ*emrAB* strains of *S*. *enterica* in the presence of nalidixic acid, novobiocin and bile salt (Fig 5). The growth of both Δ*tolC* and Δ*tolC*Δ*emrAB* was inhibited by nalidixic acid and novobiocin in the same level. In contrast, bile salt inhibited the growth of Δ*tolC*Δ*emrAB* more than Δ*tolC*, indicating TolC independent function of EmrAB in resistance to bile salt.

## Discussion

In this study, we first measured MICs of fatty acid salts with chain lengths of 6, 8, 10 and 12 carbon atoms against *S*. *enterica*. Susceptibility tests using various deletion mutants of efflux transporter genes showed no difference in susceptibility between the strains in the presence of sodium hexanoate and sodium octanoate, except for the *tolC*-deleted strains. On the other hand, in the presence of sodium decanoate and sodium dodecanoate, the changes of susceptibilities of *S*. *enterica* deletion mutants of *acrAB* and *tolC* were observed. This difference of fatty acid salts in susceptibilities might depend on the bacterial toxicity of each fatty acid salt, indicating that the fatty acid salts having the longer the chain length has more antibacterial activity. In particular, the antibacterial effect of sodium dodecanoate was more clearly demonstrated in *S*. *enterica* strain lacking *emrAB* and *tolC*.

In addition to the MIC measurements, the results of the growth assay also revealed the involvement of EmrAB in the resistance of *S*. *enterica* to sodium dodecanoate. Furthermore, the ability of EmrAB in resistance to fatty acid salts and bile salts was TolC-independent in *S*. *enterica*. This means that EmrAB can contribute to resistance to fatty acid salts and bile salts without forming a complex with TolC. The formation of the EmrAB-TolC complex is essential for the efflux of other antimicrobials [25, 26], but not for resistance against cell membrane-damaging substances such as fatty acid salts and bile salts. In the presence of fatty acid salts, the expression of *emrAB* is up-regulated, which may also contribute to the important role of EmrAB in fatty acid salts resistance in *S*. *enterica*.

A hypothesis to explain the TolC-independent function of EmrAB is that EmrAB utilizes outer membrane proteins other than TolC, or that EmrAB does function without outer membrane proteins for fatty acid and bile resistance in *S*. *enterica*. It was previously reported that MdsAB efflux system in *S*. *enterica* can utilize both MdsC and TolC outer membrane proteins to function [8]. There is no difference in sensitivity to sodium dodecanoate between the *tolC* single mutant and the *tolC mdsABC* double mutant, suggesting that MdsC does not contribute to fatty acid salts resistance modulated by EmrAB. In order to identify genes like EmrAB that make *S*. *enterica* susceptible to sodium dodecanoate by further deletion from the Δ*tolC* strain, random gene disruption mutants were generated from Δ*tolC* and screened to select sensitive strains. Ten sensitising strains were identified from approximately 3,000 mutants. In addition to *emrAB*, we found that following genes are disrupted in the sodium dodecanoate sensitive strains: *rfaP*, *rfaG* and *rfbG*, which are involved in lipopolysaccharide synthesis; *yfgL*, which is encoding an outer membrane lipoprotein; *aroK*, which is involved in amino acid biosynthesis; *rob*, a regulator gene involved in drug resistance; and *yicL*, whose function is putative permease of integral membrane protein (Table 3). It is unclear whether these genes related with fatty acid salts resistance modulated by EmrAB of *S*. *enterica*, but the mechanism by which they are involved in this resistance need to be understood in further research. The present study shows that EmrAB is involved in fatty acid salts resistance in a TolC-independent manner in *S*. *enterica*.

**Table 3:** Disrupted genes in the sodium dodecanoate susceptible mutants of *S*. *enterica*.

Gene	Gene number	Known or predicted function
*rfaP*	STM3721	Kinase that phosphorylates core heptose of lipopolysaccharide
*rfaG*	STM3722	Glucosyltransferase I involved in lipopolysaccharide synthesis
*rfbG*	STM2091	CDP glucose 4,6-dehydratase involved in lipopolysaccharide synthesis
*yfgL*	STM2520	Putative serine/threonine protein kinase encoding an outer membrane lipoprotein
*aroK*	STM3487	Shikimate kinase I involved in amino acid biosynthesis
*rob*	STM4586	Transcriptional regulator involved in drug resistance
*yicL*	STM3765	Putative permease of integral membrane protein
